# Supplementary material for: Time-resolved laser speckle contrast imaging (TR-LSCI) of cerebral blood flow response to intracranial pressure elevation
Source: J Biomed Opt. 2026 Aug 28;31(8):086007. doi: 10.1117/1.JBO.31.8.086007 (PMC13524067; doi:10.1117/1.JBO.31.8.086007)
Supplement: Supplementary file 1 [file JBO_031_086007_SD001.pdf]

Supplementary Gate-Dependent TR-LSCI Analysis Results

Additional analyses were performed to evaluate gate-dependent TR-LSCI performance across all acquired gates. Supplementary **Figs. S1-S3** present representative results from two adult rats (Rat #1 in **Fig. S1** and Rat #2 in **Fig. S2**) and the piglet (**Fig. S3**), including raw intensity images with selected cortical ROIs, BFI maps, and temporal variations in BFI and rCBF across gates. For improved visualization of cerebral hemodynamic trends, BFI and rCBF time courses were lowpass filtered prior to plotting. Supplementary **Fig. S4** compares cardiac pulsatility waveform morphology across gates. Supplementary **Table S1** evaluates the effect of gate position on HR estimation derived from rCBF signals, and Supplementary **Table S2** summarizes across-gate equivalence testing of HR estimates (TOST) in adult rats.

Figs. S1b-S1d, **Figs. S2b-S2d**, and **Figs. S3b-S3d** show BFI images across gates. Later gates (Gates #35 and #55 for Rat #1 and Rat #2; Gates #40 and #65 for the piglet) exhibit higher BFI values than the earliest gate (Gate #0), with regions of elevated perfusion becoming more pronounced at higher gate numbers. This trend is expected, as photons detected at later gates sample deeper tissue volumes, increasing the relative contribution from deeper brain regions where BFI is typically higher than in the skull. The gate dependence is more pronounced in the piglet than in the rats, likely due to the thicker piglet skull, which enhances differentiation between superficial skull and underlying brain across photon arrival times.

Note that, in addition to true depth sensitivity, the finite instrument response function (IRF) can also contribute to between-gate BFI differences through partial pathlength mixing [76]. Because IRF correction was not performed in this study, these two effects cannot be separated. Therefore, the gate selections described below were based primarily on SNR and spatial resolution,

and the between-gate BFI differences are interpreted qualitatively. A fully quantitative comparison of absolute BFI across gates will require IRF-corrected analysis.

Fig. S1e, **Fig. S2e**, and **Fig. S3e** show BFI time courses across gates. In rats, BFI magnitudes vary only modestly with gate number. In contrast, in the piglet, later gates yield higher BFI values. This likely reflects the greater contribution of deeper brain tissue at later gates in the piglet, which has a thicker skull (~2.0 mm, as measured in this study), whereas in rats, all gates predominantly sample cortical tissue due to the thinner skull (~0.5-1.0 mm) [60]. Thus, gating primarily affects tissue weighting and BFI signal magnitude rather than the timing of the underlying cerebral hemodynamic dynamics.

Fig. S1f, **Fig. S2f**, and **Fig. S3f** show rCBF time courses across gates. In rats, rCBF traces from Gates #0, #35, and #55 closely overlap across stages, indicating largely gate-independent hemodynamics. In the piglet, rCBF temporal trends are also consistent; however, the deepest gate (Gate #65) shows smaller relative changes, likely due to reduced photon counts in deeper brain.

These results support our gate selection for rCBF analysis. For rats, Gate #0 was used because rCBF trends are consistent across gates, and earlier gates provide higher SNR and spatial resolution. Moreover, the thin rat skull limits the benefit of delayed gating, as all selected gates predominantly sample cortical tissue. For the piglet, Gate #40 was selected as an intermediate gate that enhances sensitivity to brain tissue relative to superficial skull compared with Gate #0, while preserving stronger and more reliable dynamics than the deepest gate (Gate #65). Thus, Gate #40 represents a practical balance among depth sensitivity, signal robustness, and spatial resolution in thicker brain tissue.

Fig. S4 compares one-second pulsatile rCBF waveforms across gates in the rats and piglet. Cardiac pulsatility is preserved across all gates, with similar waveform morphology, peak timing,

and beat-to-beat periodicity, and only modest amplitude differences. These results indicate that TR-LSCI maintains sufficient temporal fidelity to capture pulsatile rCBF while enabling depth-sensitive gated measurements. Importantly, rCBF responses used to assess cerebral autoregulation remain consistent across gates.

Table S1 demonstrates highly consistent HR estimates across gates within each animal, with only minimal variation. **Table S2** presents statistical equivalence testing results across gates in adult rats using the two one-sided tests (TOST) framework described in the main manuscript. The results confirm measurement equivalence, with all tests yielding $p < 0.001$. Statistical equivalence testing was not performed for the neonatal piglet due to the single-subject design. Together, these findings indicate that FFT-derived HR estimates are not materially affected by gate position, further supporting the preservation of cardiac pulsatility in time-resolved TR-LSCI measurements.

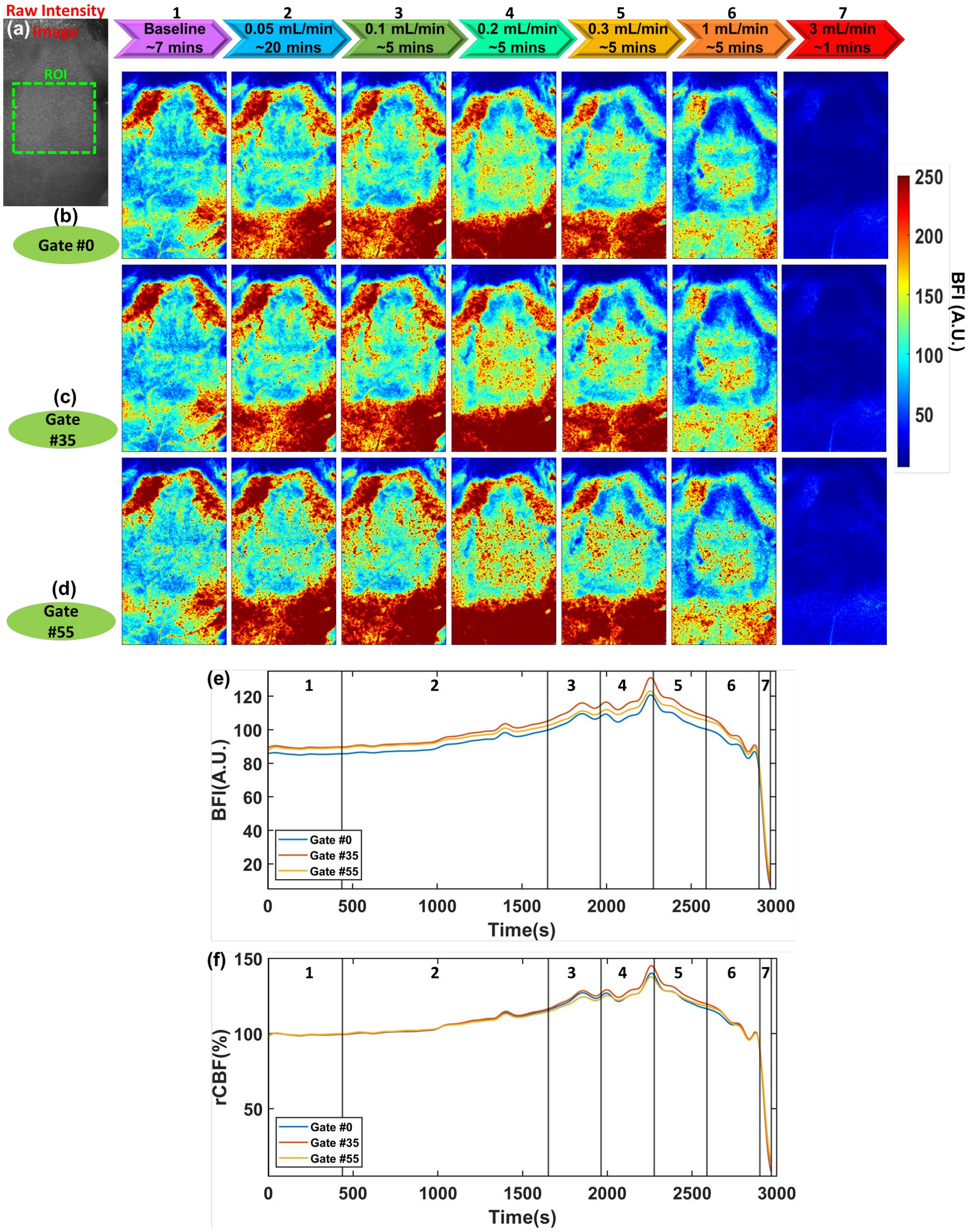

Fig. S1: Representative TR-LSCI results from Rat #1 demonstrating depth dependent CBF responses during intracranial infusion. (a) Raw intensity image showing the cortical ROI used for time-course data analysis. **(b-d)** BFI maps acquired at Gates #0, #35, and #55, respectively, during baseline and successive infusion stages (0.05, 0.1, 0.2, 0.3, 1, and 3 mL/min), illustrating gate-dependent variations in BFI magnitude and spatial blood flow distribution. In general, later gates exhibited higher BFI values while preserving similar overall cortical perfusion patterns across physiological conditions. **(e)** Corresponding ROI-based BFI time courses derived from the selected cortical region for Gates #0, #35, and #55. BFI magnitudes across gates were similar, with modest differences observed and no consistent monotonic increase with gate number. All time courses showed similar temporal responses during baseline, ICP elevation, and terminal decline. **(f)** Corresponding rCBF time courses for Gates #0, #35, and #55. The rCBF responses closely tracked one another throughout the experiment despite gate-dependent differences in BFI values, indicating consistent cerebral hemodynamic trends across gates.

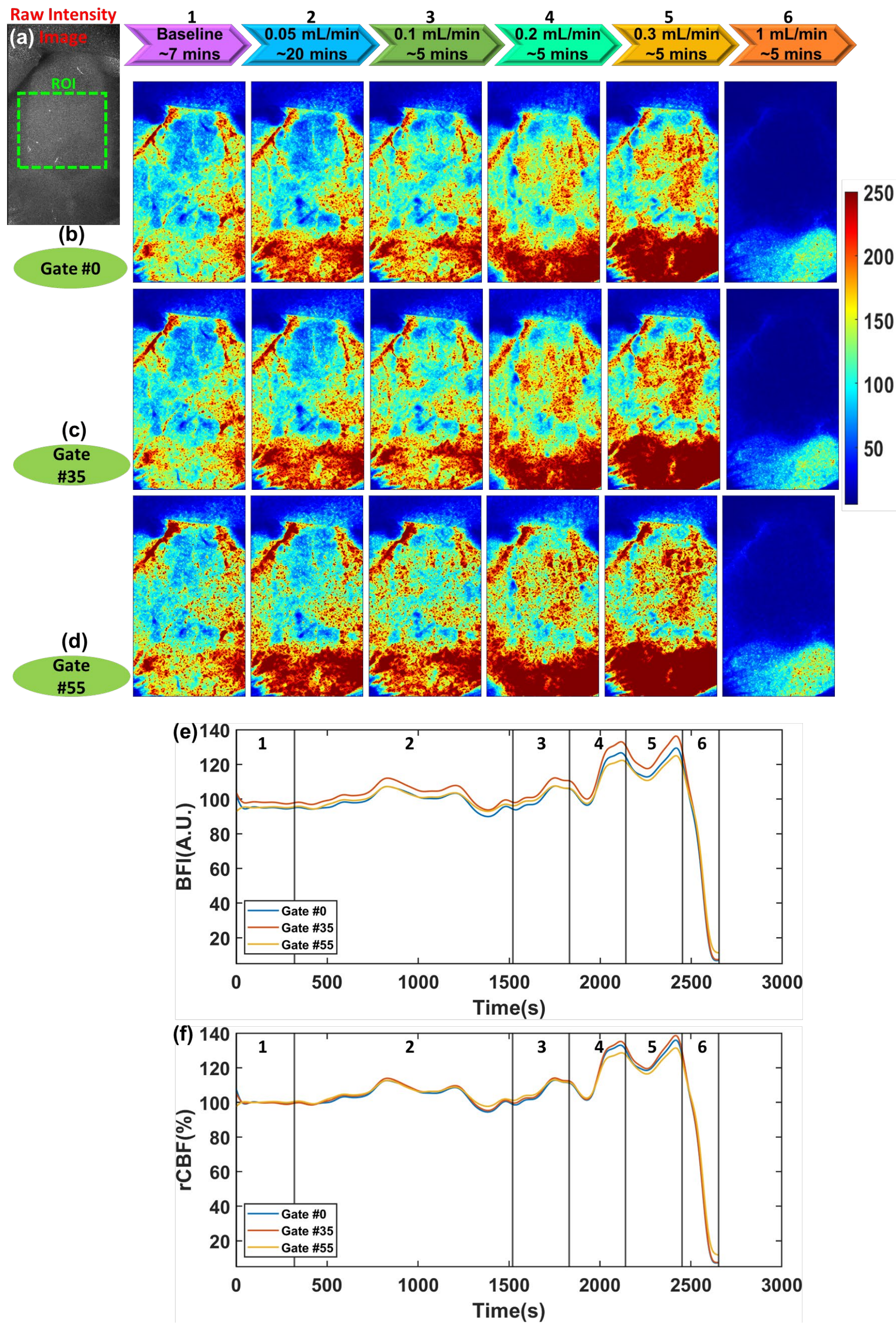

Fig. S2: Representative TR-LSCI results from Rat #2 demonstrating depth dependent CBF responses during intracranial infusion. (a) Raw intensity image showing the cortical ROI used for time-course data analysis. **(b-d)** BFI maps acquired at Gates #0, #35, and #55, respectively, during baseline and successive infusion stages (0.05, 0.1, 0.2, 0.3, and 1 mL/min), illustrating gate-dependent variations in BFI magnitude and spatial blood flow distribution. In general, later gates exhibited higher BFI values while preserving similar overall cortical perfusion patterns across physiological conditions. **(e)** Corresponding ROI-based BFI time courses derived from the selected cortical region for Gates #0, #35, and #55. BFI magnitudes across gates were similar, with modest differences observed and no consistent monotonic increase with gate number. All time courses showed similar temporal responses during baseline, ICP elevation, and terminal decline. **(f)** Corresponding rCBF time courses for Gates #0, #35, and #55. The rCBF responses closely tracked one another throughout the experiment despite gate-dependent differences in BFI values, indicating consistent cerebral hemodynamic trends across gates.

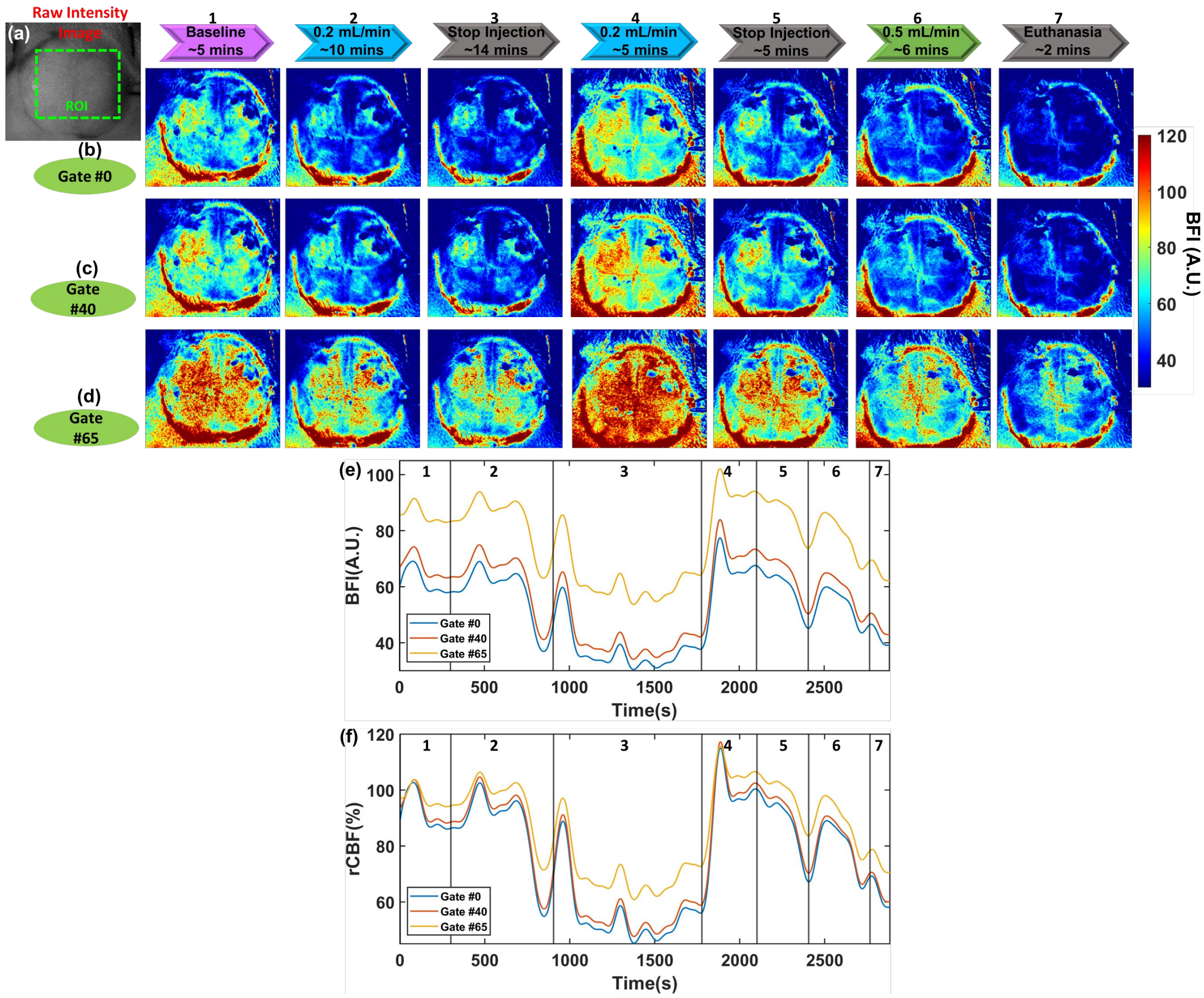

Fig. S3: Representative TR-LSCI results from the piglet demonstrating depth dependent CBF responses during intracranial infusion. (a) Raw intensity image showing the cortical ROI used for time-course data analysis. **(b-d)** BFI maps acquired at Gates #0, #40, and #65, respectively, during baseline and repeated infusion and pause cycles (0.2 mL/min, stop injection, 0.2 mL/min, stop injection, 0.5 mL/min), followed by the terminal phase, illustrating gate-dependent variations in BFI magnitude and spatial blood flow distribution through the intact skull. In general, later gates exhibited higher BFI values, with Gate #65 showing the strongest BFI signal and more pronounced gate-dependent contrast. **(e)** Corresponding BFI time courses derived from the selected cortical region for Gates #0, #40, and #65. Although the absolute BFI magnitude increased with gate number, all gates demonstrated similar overall temporal responses during infusion, stop-injection, recovery, and terminal phases. **(f)** Corresponding rCBF time courses for Gates #0, #40, and #65. The overall rCBF trends remained consistent across gates; however, the deepest gate (Gate #65) exhibited smaller rCBF changes than Gates #0 and #40, suggesting reduced dynamic sensitivity at the latest gate despite higher BFI values.

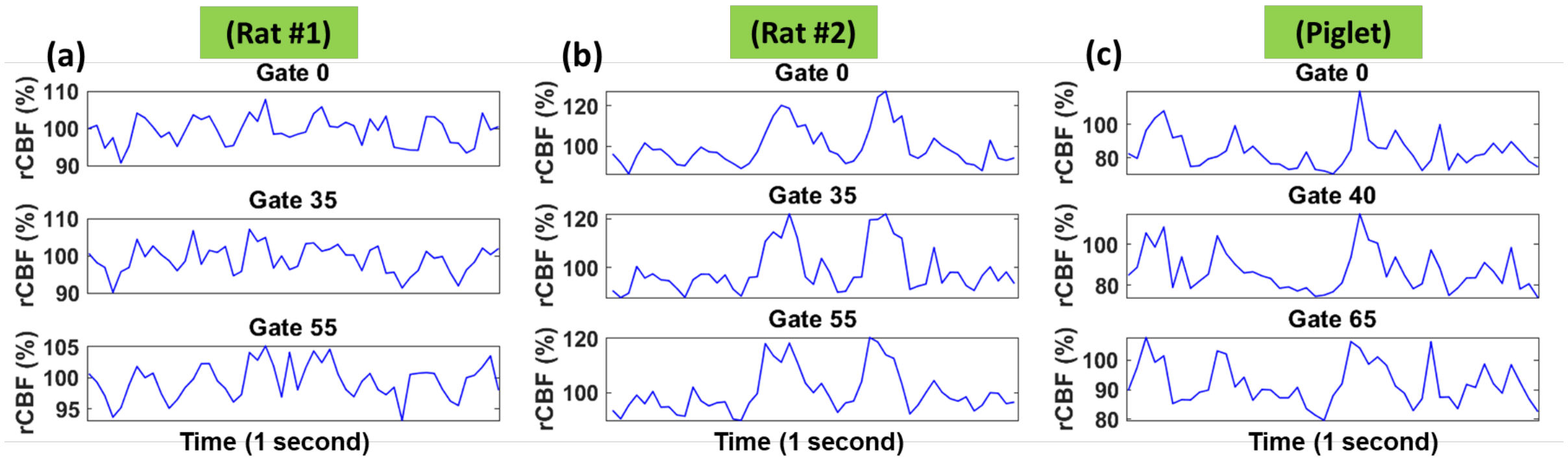

Fig. S4: One-second pulsatile rCBF waveforms across gates in three representative animals: (a) Rat #1, (b) Rat #2, and (c) Piglet. rCBF signals were extracted from Gates #0, #35, and #55 for rats or Gates #0, #40, and #65 for the piglet. Cardiac pulsatility was preserved across all analyzed gates, with similar waveform morphology, peak timing, and beat-to-beat periodicity. Only modest gate-dependent differences in pulsation amplitude were observed.

Table S1: Baseline heart rates (mean ± standard deviation, Hz) across gates derived from rCBF

Subject	Gate #0 (Rat)/Gate #0 (Piglet)	Gate #35 (Rat)/Gate #40 (Piglet)	Gate #55 (Rat)/Gate #65 (Piglet)
Rat #1	7.24 ± 0.05	7.24 ± 0.05	7.24 ± 0.05
Rat #2	6.87 ± 0.12	6.87 ± 0.10	6.86 ± 0.14
Rat #3	7.14 ± 0.12	7.15 ± 0.05	7.14 ± 0.10
Rat #4	8.41 ± 0.38	8.41 ± 0.37	8.42 ± 0.37
Piglet	3.21 ± 0.03	3.21 ± 0.03	3.21 ± 0.03

Table S2: Across-gate equivalence testing of HR estimates (TOST) in rats (n = 4)

Comparison	90% CI (Hz)	p (> -0.25 Hz)	p (< +0.25 Hz)	Equivalence
$rCBF_{Gate\ \#0}\ vs\ rCBF_{Gate\ \#35}$	[-0.0084, 0.0034]	0.0000	0.0000	Yes
$rCBF_{Gate\ \#0}\ vs\ rCBF_{Gate\ \#55}$	[-0.0096, 0.0096]	0.0000	0.0000	Yes
$rCBF_{Gate\ \#35}\ vs\ rCBF_{Gate\ \#55}$	[-0.0088, 0.0138]	0.0000	0.0000	Yes
